# Unraveling the complexity of cognitive impairment following high-altitude exposure: from preclinical animal models to human organoids

**DOI:** 10.3389/fnins.2025.1679858

**Published:** 2025-11-17

**Authors:** Hong Gong, Yu-Xin Liu, Qing-Lu Xiaoluo, Mei-Feng Gong, Zhi Liu, Sheng-Ru Wu, Zhen-Yang Chen, Tian-Yao Liu, Jing-Hui Zhao, Lian Wang, Xiao-Tang Fan, Hai-Wei Xu

**Affiliations:** 1 Southwest Eye Hospital, Southwest Hospital, Third Military Medical University (Army Medical University), Chongqing, China; 2 Department of Military Cognitive Psychology, School of Psychology, Third Military Medical University (Army Medical University), Chongqing, China; 3 Battalion 5 of the Cadet Brigade, School of Basic Medical Sciences, Third Military Medical University (Army Medical University), Chongqing, China; 4 Plateau Brain Science Research Center, Tibet University, Lhasa, China

**Keywords:** cognition, high-altitude, pathophysiological mechanisms, animal models, *in vitro* models, brain organoids

## Abstract

Long-term exposure to high-altitude hypobaric hypoxia has a detrimental effect on cognitive function. These effects are dependent on multiple factors, including altitude, exposure duration, pre-acclimatization conditions, psychological traits, and individual differences. Existing studies have investigated pathogenesis, epidemiology, and interventions for hypobaric hypoxia-associated cognitive impairment based on population studies and preclinical models. The underlying pathophysiological mechanisms involve energy metabolism, neuronal autophagy, oxidative stress, inflammatory response, gut microbiota imbalances, and genetic susceptibility. However, no consensus has been reached on the most important mechanisms and most recommended animal models, and no standard effective interventions are currently available. This review aims to provide new insights and research perspectives for cognitive impairment following high-altitude exposure. By systematically summarizing the pathophysiological mechanisms of hypobaric hypoxia-associated cognitive impairment, we comprehensively compare animal models for studying high-altitude-induced cognitive decline using three paradigms, i.e., mild, moderate, and extreme high-altitude exposure. Additionally, we introduce various *in vitro* models, including pluripotent stem cells and brain organoids, which can be used to evaluate the potential mechanisms and therapies. Furthermore, we analyze the challenges in current studies and propose key research directions for future work.

## 1 Introduction

In geography, a relatively extensive highland exceeding 500 m in altitude is called a plateau. In medicine, however, a plateau is defined as a broad area or elevated region above 3,000 m that induces significant biological effects. More recently, people believe this altitude standard should be reduced to 2,500 m above sea level, where about 400 million people live (León-Velarde et al., 2005). In China, nearly 80 million inhabitants reside across four high plateaus: the Qinghai-Xizang Plateau, Yunnan-Guizhou Plateau, Inner Mongolia Plateau, and Loess Plateau (León-Velarde et al., 2005). Additionally, an increasing number of people travel to high-altitude locations for work or tourism. The high-altitude plateau environment is characterized by low atmospheric pressure, low oxygen levels, substantial diurnal temperature variations, and intense ultraviolet radiation, which may adversely impact physiological and psychological wellbeing in both residents and visitors.

Oxygen deficiency can reduce oxygen levels in the blood and induce hypoxemia. With the increase in altitude, atmospheric pressure decreases, resulting in a reduction of the partial pressure of ambient oxygen (PaO_2_; Sevik et al., 2025). At sea level, the typical arterial PaO_2_ of arterial blood ranges from 95% to 100% in healthy individuals. An epidemiological study of 6,289 healthy subjects shows that SpO_2_ decreases with rising altitude, particularly at elevations exceeding 2,500 m (Rojas-Camayo et al., 2018). The median interquartile range (IQR) of SpO_2_ reduces from 96 (95–97) at 2,500 m to 81 (78–84) at 5,100 m (Rojas-Camayo et al., 2018). Hypoxemia occurs when arterial PaO_2_ falls below 95%, and the clinical severity correlates with lower PaO_2_ levels. Acute exposure to a high-altitude environment can trigger various high-altitude illnesses (HAIs), while chronic mountain sickness (CMS) may affect both high-altitude immigrants and long-term resident populations worldwide (Gatterer et al., 2024).

As one of the most active organs, the brain consumes about 20% of the body's oxygen supply. In addition to organic brain diseases, such as HACE, hypobaric hypoxia can also trigger functional brain diseases, including mental diseases and neurodegenerative diseases. For decades, scientists and physicians have thoroughly investigated the effects of high-altitude hypoxia on cognitive function (McMorris et al., 2017), including attention, episodic memory, executive function, and information processing (Roach et al., 2014). Since cognitive abilities are brain-based skills that enable us to complete daily tasks and work at various levels of difficulty, hypoxia-induced cognitive deficits may impair performance in high-altitude areas. A systematic review with 38 original articles indicates that acute exposure to hypobaric hypoxia within minutes or hours can lead to extensive cognitive impairments (Post et al., 2023). The pooled results reveal 83% of participants exhibit delayed recognition, and 50% of participants are disturbed by visual/spatial delayed recognition (Post et al., 2023). Another recent systematic review and meta-analysis evaluated the long-term effects of hypoxic exposure on cognition based on 49 studies with 6,191 individuals (Su et al., 2024a). The findings are similar yet more complex compared to the acute exposure to hypobaric hypoxia. Some types of cognitive tasks, especially psychomotor and long-term memory tasks, are the most vulnerable. Whereas, a few cognitive abilities, including perceptual processes and inhibitory control, may not be affected significantly by hypobaric hypoxia (Su et al., 2024a).

To date, only one drug, acetazolamide, has been approved by the Food and Drug Administration for the prophylaxis of acute mountain sickness (Low et al., 2012). Although studies have demonstrated the adverse effects of hypobaric hypoxia on cognition, the underlying pathology is still largely unknown. Regarding cognitive health in high-altitude environments, no approved drugs are currently available. Multiple mediators, such as altitude, exposure duration, and individual differences, contribute to the modulation of cognitive responses, making it complicated when uncovering the underlying mechanisms. Preclinical models provide the possibility to further reveal the biochemical and molecular mechanisms of cognitive decline after exposure to hypobaric hypoxia. The present review aims to summarize the current knowledge of the mechanisms of hypobaric hypoxia-induced cognitive impairment and elucidate the advances of relevant preclinical models.

## 2 Potential mechanisms of cognitive changes after exposure to high-altitude hypoxia

**Figure 1:**
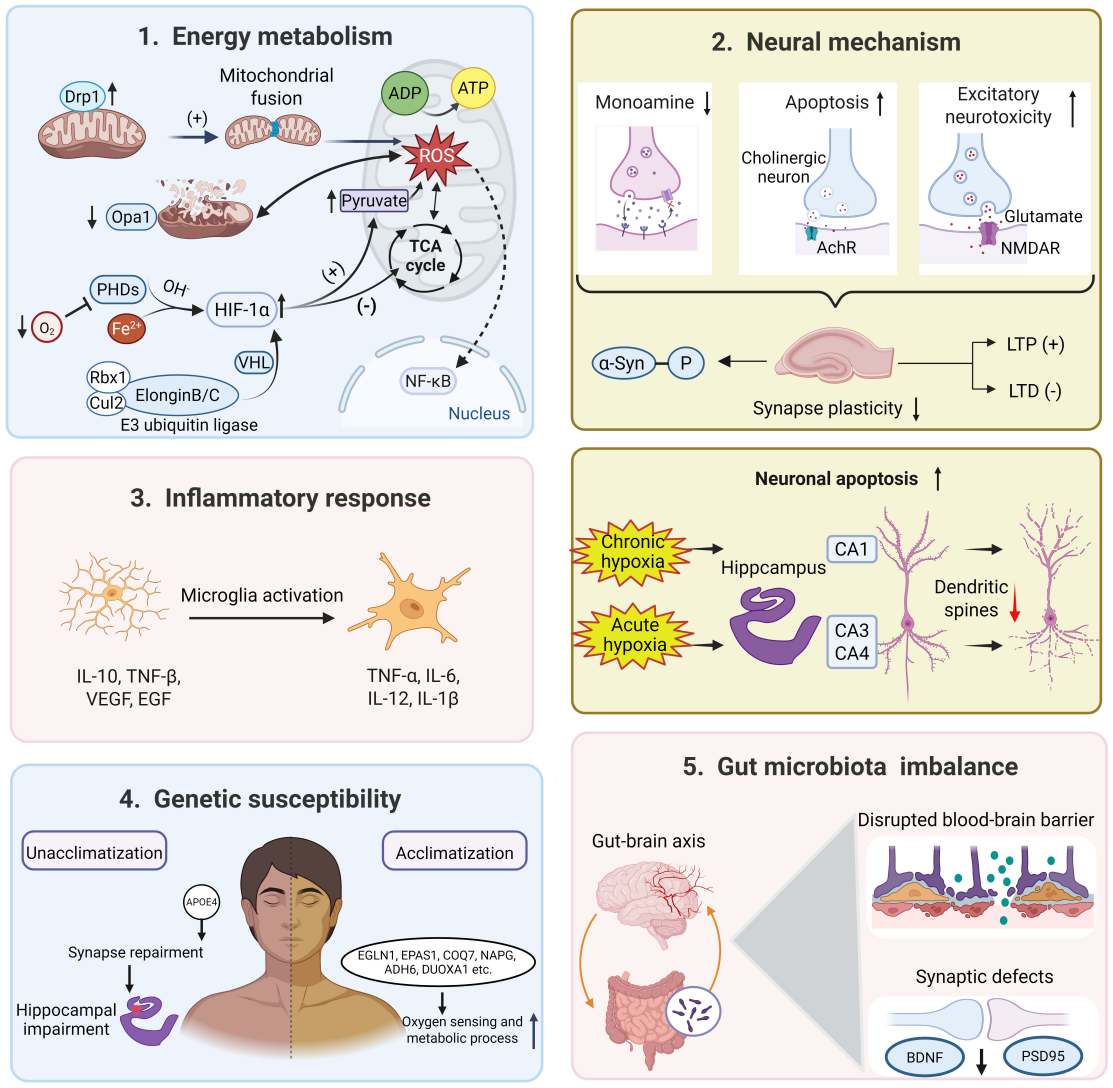
The potential mechanisms underlying cognitive dysfunction induced by high-altitude hypobaric hypoxia. Image was created with BioRender.com with permission.

Hypoxia affects brain function and leads to cognitive and behavioral deficits (Chattopadhyaya et al., 2025). As altitude increases and oxygen levels decrease, various symptoms occur, including headache, fatigue, dyspnea, and potentially life-threatening conditions in severe cases. Therefore, investigating the mechanisms behind hypoxic diseases is crucial for ensuring the safety and health of individuals in high-altitude regions. De Bels et al. (2019) demonstrated that acute exposure to hypobaric hypoxic conditions resulted in significant decreases in cerebral blood flow index [CBFi = MAP × 10/1.47 × PI, PI = (velocity systolic-velocity diastolic)/mean velocity, MAP = mean arterial pressure] and blood oxygen saturation, along with an increase in heart rate. These physiological changes are helpful to increase the oxygen uptake, but the pathological changes are detrimental and may even trigger chronic mountain sickness (Villafuerte et al., 2022). In terms of cognitive function, it is widely accepted that chronic exposure to the plateau environment is a robust risk factor for cognitive decline in plateau immigrants (Sharma et al., 2019). Multiple pathophysiological mechanisms may be implicated in the development of hypoxia-induced cognitive decline. For instance, hypobaric hypoxia reduces the expression of synaptic molecules and interrupts the synaptic plasticity, which disrupts the limbic region-based fear memory (Kumari et al., 2018). As shown in Figure 1, cognitive impairment after hypoxia exposure involves dysfunctions in energy metabolism, neuronal apoptosis, inflammatory response, genetic susceptibility, as well as gut microbiota imbalance.

### 2.1 Energy metabolism

It is well-known that oxygen is critical for cellular metabolism. Thus, almost all the physiological functions are inevitably affected under the hypoxic conditions encountered at high altitude (Richalet et al., 2024). Hypobaric hypoxia drives a metabolic shift to glycolysis rather than oxidative phosphorylation, leading to excessive lactate production and PKM2 lactylation, which aggravates vascular endothelial dysfunction (Zhang Y. et al., 2025). Regional cerebral blood flow and oxygen delivery (CDO_2_) is a robust marker for the level of oxygen in brain tissue. In acute hypoxia, the middle cerebral artery (MCA) CDO_2_ and posterior cerebral artery (PCA) CDO_2_ are reduced during the central executive and non-executive tasks (Ando et al., 2023). The MCA is responsible for oxygen delivery to most areas of the frontal, parietal, and temporal lobes and controls motor, sensory, and many higher cognitive functions. The PCA is responsible for the blood supply of the occipital lobe, medial temporal lobe, part of the thalamus, and midbrain, and is related to sensory integration functions such as vision and memory (Ando et al., 2023). Consequently, reduced oxygen delivery to the MCA and PCA is significantly associated with decreased accuracy in working memory (Ando et al., 2023).

The hippocampus plays a key role in learning, memory, navigation, and cognition, and has been studied extensively. The hippocampus, especially CA1 and CA3, is susceptible to hypoxia, and abnormal vascular regulation in it may lead to spatial memory impairment (Johnson, 2023). In an albino rat model of hypobaric hypoxia-induced oxygen deficiency, researchers have even revealed that the hippocampus is more susceptible to histometric changes than the cerebellar cortex and striatum under chronic hypoxia (Shushanyan et al., 2023). The hypoxic response of the MCA and PCA in high-altitude environments is a “double-edged sword”—dilation in the acute phase can compensate for oxygen supply. However, abnormal long-term regulation can lead to insufficient oxygen delivery in the hippocampus and posterior circulation regions (Sadeghzadeh et al., 2023). These vascular injuries and neurovascular coupling disorders contribute to the dysfunction of oxygen transport and energy conversion, ultimately leading to cognitive dysfunction.

Long-term hypoxia could be the outcome of energy failure and mitochondrial impairment. A glucose deficit has been found in the entorhinal cortex and parietal lobes in patients with mild cognitive impairment (Cerasuolo et al., 2023). At the cellular level, hypoxia results in energy metabolism changes in both neurons and astrocytes (Zhang et al., 2022). The discovery of hypoxia-inducible factors (HIFs) is a crucial marker for cells to respond to changes in oxygen levels. Under normoxic conditions, HIFα subunits undergo polyubiquitination mediated by the pVHL-elongin BC-CUL2 complex (hereafter designated as the VHL complex), accelerating the proteasomal degradation (Semenza, 2012). Hypoxia inhibits the hydroxylation of HIFα subunits, thereby preventing their ubiquitin-mediated proteasomal degradation. Consequently, HIFα subunits heterodimerize with HIF1β to form transcriptionally active complexes (Lee et al., 2020), and then inhibit oxygen-consuming processes and maintain energy production through reprogramming central carbon metabolism (Ma et al., 2025).

Mitochondria are known as the “energy factories” of cells and the key organelles for sensing oxygen. In mitochondria, oxygen is consumed by oxidative phosphorylation to produce ATP and provide energy for cells. Hypoxia stress causes depolarization of mitochondrial membrane potential, disrupts the process of mitochondrial fusion and fission, and reduces the stability and oxidative phosphorylation of electron transport chain (ETC) complex proteins (Fuhrmann and Brüne, 2017). In the brain, high-altitude hypoxia promotes mitochondrial fission of neurons from the hippocampus and cortex, accompanied by abnormal expressions of mitochondrial dynamics-related proteins (Huan et al., 2023). For instance, a marker of mitochondrial fusion, Opa1, is downregulated, while Drp1 and P-Drp-ser616 (markers of mitochondrial fission) are upregulated in hypoxic HT22 hippocampal neuronal cells (Huan et al., 2023). Moreover, hypoxia accelerates mitophagy through the autophagosome-lysosome pathway by activating mitophagy receptors BNIP3, FUNDC1, and BNIP3L (Liu et al., 2012). Besides mitophagy, a recent finding shows that hypoxia can reprogram normal tubular mitochondria into large spherical mitochondria and engulf lysosomes to induce mitochondrial degradation (Hao et al., 2023). The hypoxia increases ROS production and disrupts energy metabolism in dysfunctional mitochondria, leading to an energy supply crisis (Scharping et al., 2021).

At the molecular level, transcriptome data have identified thousands of differentially expressed genes (DEGs) related to hypoxia. The enrichment analysis based on these DEGs converges on energy metabolism, including the oxidative phosphorylation (OXPHOS) pathway and the tricarboxylic acid (TCA) cycle (Gao et al., 2023). AMP-activated protein kinase (AMPK) and mechanistic target of rapamycin complex 1 (mTORC1) serve as pivotal regulators of cellular energy homeostasis, orchestrating metabolic responses through the direct sensing of intracellular ATP and nutrients (e.g., glucose, amino acids). AMPK is directly activated by AMP, a near-equilibrium ATP buffer system that catalyzes the interconversion of 2 ADP molecules into ATP and AMP. Thus, the AMP/ATP ratio exhibits quadratic dependence on the ADP/ATP ratio. Under ATP-depleting conditions, activated AMPK orchestrates metabolic reprogramming to restore energy homeostasis through promoting catabolic ATP production and suppressing energy-consuming anabolic processes (Chun and Kim, 2021). AMPK activation has been reported in various tissues and cell types under hypoxia. AMPK activation within the erythrocyte ADORA2B-BPGM axis is essential for mitigating hypoxia-induced cognitive decline via promoting metabolic reprogramming (Qiang et al., 2021). As a central metabolic hub, mTORC1 exerts dual control over lipogenesis and one-carbon metabolism regulation (Qiang et al., 2021). Hypoxia-mediated mTORC1 inhibition occurs primarily via AMPK activation, with parallel direct regulation through the DNA damage/development-regulated protein REDD1/DDIT4 (Sofer et al., 2005). Targeting the AMPK-mTOR-HIF1α pathway may be a candidate therapy to mitigate cognitive deficits under the condition of Alzheimer's disease and high-altitude exposure (Su et al., 2025).

### 2.2 Neural mechanism

#### 2.2.1 Synaptic and structural plasticity

The high-altitude exposure not only affects the neural structure but also disrupts neural function. After synthesizing MRI data from the healthy human brain structure, a systematic review and meta-analysis have found no significant differences in global gray matter, white matter, or CSF volumes, or in frontal/temporal lobe FA between high-altitude and low-altitude groups. However, significant localized structural alterations (GM volume and FA) indicative of neuroadaptation were identified (Luo et al., 2023). In addition, previous evidence has uncovered that severe hypobaric hypoxia (>5,500 m) could induce irreversible neuron apoptosis in the CA3, CA4, and dentate gyrus of the hippocampus (Chen et al., 2017), which would be an essential factor in hypobaric hypoxia-induced cognitive impairment. Furthermore, exposure to hypobaric hypoxia leads to a reduction in dendritic spine density alongside an increase in spine neck length within the basal and apical dendrites of hippocampal CA1 neurons in mice (Zhou et al., 2021).

Synapses are the connecting units that allow nerve impulses to be transmitted between two neurons or between a neuron and a muscle through motor endplates. In the mammalian central nervous system, the chemical synapse is the predominant functional synapse, where neural signals are transduced via voltage-gated calcium channel activation, triggering vesicular exocytosis of neurotransmitters. Perinatal hypoxia in mice disrupts microtubule dynamic stability via Atoh1 overexpression, resulting in delayed neuronal migration, aberrant synaptogenesis, and impaired cognitive function in adulthood (Cai et al., 2024). Chronic hypoxia (13% O_2_, 7–14 days) also induces abnormal phosphorylation of α-synuclein at Ser129 (α-Syn p-S129), promotes its aggregation, and impairs synaptic integrity in the hippocampus. Therefore, hypoxia poses a significant threat to synaptic integrity and cognitive function, particularly targeting hippocampal circuits critical for memory. Synaptic plasticity, the activity-dependent modification of synaptic strength, constitutes the primary cellular substrate for learning and memory. The dynamical synaptic connections are essential for diverse cognitive functions, including memory reinforcement, relearning, and associative memory formation (Bin Ibrahim et al., 2022).

#### 2.2.2 Neurotransmitter and neuropeptide dysregulation

Neurotransmitters are a class of endogenous signaling molecules that act as “messengers” in synaptic transmission. In a physiological state, a delicate balance between inhibition (GABAergic signaling) and excitation (glutamatergic signaling) is the functional foundation for normal cognition. Hypoxia, particularly at high altitudes, induces significant dysregulation in the glutamatergic system, playing a central role in the pathogenesis of associated cognitive impairments. Acute hypoxia triggers excessive glutamatergic neuronal excitability and even evokes excitotoxicity. Therefore, synaptic plasticity damage and neuronal apoptosis may directly contribute to cognitive dysfunction (Jiao et al., 2024). Chronic hypoxia induces a substantial reduction in hippocampal glutamatergic synapse density and a decrease in glutamate/glutamine (Glu/Gln) concentration, suggesting a weakened excitatory neurotransmission. These changes are strongly correlated with deficits in learning, memory, attention, and alertness (Lin et al., 2011).

Moreover, chronic hypoxia has been shown to reduce tyrosine hydroxylase activity and dopamine synthesis, which further impairs spatial memory (Zhong et al., 2024). Mechanistically, HIFα upregulation disrupts monoamine metabolism (Mohamed et al., 2025), while VMAT2 dysfunction compromises synaptic vesicle loading and neurotransmitter release (Wu et al., 2025). Altered monoamine oxidase (MAO) activity accelerates degradation, with abnormal metabolite ratios (e.g., elevated DOPAC/DA) correlating with memory deficits (Chen et al., 2023). These perturbations converge to disrupt prefrontal BDNF-CREB signaling (Wang et al., 2022), directly impairing executive function, attention, and emotional regulation. Critically, serotonergic dysfunction enhances stress susceptibility (Naoi et al., 2025), and norepinephrine/epinephrine imbalances correlate with cognitive decline in hypoxic conditions (e.g., OSA; Ji et al., 2025). Future studies are needed to explore the role and interaction of monoamine neurotransmitter systems under hypobaric hypoxia conditions.

Hypoxia at high altitudes also leads to significant dysregulation of the cholinergic system. Hypobaric and chronic intermittent hypoxia (CIH) trigger apoptosis of basal forebrain cholinergic neurons, which project to hippocampal and cortical regions essential for learning, memory, and attention, and disrupt cholinergic neural circuits (Tang et al., 2020; Northington et al., 2022). The resultant degradation of cholinergic pathway integrity, as evidenced by early white matter disruption in hypoxic conditions and quantified by biomarkers like the Cholinergic Pathway Hypersignal Scale (CHIPS), directly correlates with cognitive decline in disorders ranging from obstructive sleep apnea to Moyamoya disease (Nemy et al., 2023; Xu et al., 2024). Critically, basal forebrain atrophy and cholinergic connectivity loss are associated with subjective cognitive decline, suggesting neurodegenerative-like pathophysiology (Rodriguez-Hernandez et al., 2024).

Last but not least, hypoxia at high altitudes induces significant dysregulation of neuropeptide signaling, a critical contributor to cognitive impairment through dual mechanisms of pathway disruption and compromised neuroprotection. Hypoxia triggers aberrant expression of neuropeptides like cholecystokinin (CCK)-where altered levels correlate with synaptic damage and cognitive decline in neurodegeneration-and disrupts key signaling pathways: Neuropeptide W impairs the PKA/CREB cascade via GPR7/8 receptors, inhibiting protein glycosylation essential for cognitive function (Nguyen et al., 2024), while melanocortin dysregulation through MC3R compromises the PI3K/AKT survival pathway, exacerbating neuronal apoptosis. Conversely, protective neuropeptides such as pituitary adenylate cyclase-activating polypeptide (PACAP) and vasoactive intestinal peptide (VIP) demonstrate neurotrophic capacities by activating cAMP-mediated anti-apoptotic mechanisms (Solés-Tarrés et al., 2020); however, hypoxia overwhelms these safeguards by suppressing their survival pathways (PI3K/AKT, BDNF, MAPK; Chen et al., 2013). This imbalance mediates synaptic modulation and cripples endogenous neuroprotective responses, converging to disrupt synaptic efficacy, neuronal viability, and ultimately higher-order cognitive processing, positioning neuropeptide dysregulation as a pivotal therapeutic target in hypoxia-induced cognitive deficits.

Previous evidence suggests that various cognitive domains are differently affected by high altitude exposure (Su et al., 2024a). After long-term exposure to high altitude, psychomotor function and long-term memory are most vulnerable. Executive function and language skills are moderately affected. Interestingly, perceptual processes and problem-solving abilities are still resilient. Such selective cognitive adaptation may result from impairing motor cortex-related functions and optimizing prefrontal cortex-related functions (Su et al., 2024a). However, in addition to the traditional behavioral tests for learning and memory, reliable neural mechanisms need to be further explored. On the one hand, more functional neuroimaging evidence is needed to determine the functional connection and neuroplasticity following high altitude exposure. On the other hand, specific experimental paradigms of cognition are necessary to uncover the neuropathological mechanism. For instance, executive function can be specifically assessed by a touchscreen operant platform in rodents.

### 2.3 Oxidative stress and inflammatory response

Acute or chronic hypoxia induces profound oxidative stress primarily by disrupting mitochondrial function. This disruption leads to an excessive generation of reactive oxygen species (ROS) due to electron transport chain impairment and reduced ATP production, creating an energy crisis within neurons. The resultant ROS overproduction triggers cascading oxidative damage, including mitochondrial membrane dysfunction, disruption of neurotransmitter synthesis and release (e.g., monoamines like 5-HT and dopamine, and imbalance between glutamate and GABA), and impairment of synaptic plasticity (Soren et al., 2024). Critically, mitochondrial oxidative stress exacerbates energy metabolism disorders and can directly induce neuronal apoptosis, forming a core mechanism underlying hypoxia-associated neuronal damage and cognitive decline (Lv et al., 2023). This is evidenced by the suppression of protective pathways like BDNF/PI3K/AKT and Nrf2-mediated antioxidant defenses (Yao et al., 2022).

Neuroinflammation is one of the key hallmarks after hypoxia exposure, characterized by the activation of microglia and astrocytes. Alterations in the neuroimmune microenvironment lead to microglial polarization toward a pro-inflammatory (M1) phenotype and the subsequent release of cytokines (e.g., IL-6, IL-1β) and markers like galectin-3 (Wang et al., 2023). Hypoxia further activates key inflammatory pathways, notably the NLRP3 inflammasome and NF-κB signaling, amplifying the production of pro-inflammatory mediators (He et al., 2025). Metabolites such as TMAO can exacerbate this response by inhibiting SIRT1 (Deng et al., 2022). This neuroinflammatory state disrupts synaptic integrity and neurotransmitter balance, promotes excitotoxicity, and synergizes with oxidative stress pathways. Importantly, hypoxia and neuroinflammation exhibit a bidirectional relationship, creating a vicious cycle that exacerbates neuronal damage and apoptosis, particularly in the hippocampus, thereby contributing significantly to cognitive impairment.

Collectively, hypoxia-induced oxidative stress and neuroinflammation represent pivotal, interconnected pathological mechanisms driving cognitive dysfunction under high-altitude conditions. Oxidative stress causes direct neuronal damage, energy failure, and neurotransmitter dysregulation, while neuroinflammation amplifies tissue injury and disrupts synaptic function. Their synergistic actions, through pathways like mitochondrial dysfunction, NLRP3 inflammasome activation, impaired neurotrophic support (e.g., BDNF), and excitotoxicity, culminate in synaptic structural damage, neuronal apoptosis, and impaired hippocampal plasticity. Clinical observations linking inflammatory markers and metabolic changes in red blood cells to cognitive deficits in high-altitude residents further underscore the central role of these mechanisms in mediating the detrimental cognitive effects of chronic hypoxic exposure.

### 2.4 Gut microbiota

Accumulating evidence has shown that high-altitude hypoxia induces significant dysbiosis in the gut microbiota, characterized by reduced abundance of beneficial genera (e.g., Ligilactobacillus, Muribaculum) and enrichment of pathogenic bacteria such as Clostridium species, which demonstrate high predictive value for cognitive impairment (Bai et al., 2022). This dysbiosis compromises intestinal barrier integrity, evidenced by mucosal exfoliation, downregulation of tight junction proteins, and increased intestinal permeability (Shang et al., 2025). Consequently, bacterial endotoxins (e.g., LPS) translocate into systemic circulation, while hypoxia and gut-derived inflammatory factors synergistically disrupt the blood-brain barrier (BBB; Zhou et al., 2025). This cascade permits peripheral inflammatory mediators to infiltrate the CNS, triggering neuroinflammation and directly impairing hippocampal synaptic plasticity, manifested as reduced expression of BDNF and PSD-95, neuronal activity imbalance, and disrupted functional connectivity in spatial memory-related regions (prefrontal cortex; Chen et al., 2025; Zhang J. et al., 2023).

The brain-gut axis, a bidirectional communication network involving neural, endocrine, and immune pathways, serves as a critical conduit for hypoxia-induced cognitive dysfunction. Hypoxia exacerbates dysregulation of the hypothalamic-pituitary-adrenal (HPA) axis, further amplifying neuroinflammation and synaptic damage, while gut microbiota-derived metabolites (e.g., via cytochrome P450/drug transporters) indirectly modulate brain function (Bai et al., 2022). Crucially, fecal microbiota transplantation (FMT) in hypoxic murine models partially reverses working memory deficits, confirming the causal role of microbial dysbiosis in cognitive impairment (Zhao et al., 2022). The above evidence highlights the gut-brain axis pathway as a fundamental mechanism linking high-altitude hypoxia to cognitive decline. More importantly, clinical intervention studies with FMT and/or identified microflora (e.g., Clostridium species, Lactobacillus johnsonii YH1136) are essential to confirm efficacy and safety for high-altitude participants.

### 2.5 Genetic susceptibility

Genetic polymorphisms significantly influence individual susceptibility to high-altitude hypoxia-induced cognitive impairment. Variations in genes regulating the HIF pathway directly modulate hypoxia sensitivity and antioxidant capacity (e.g., SOD activity), while APOE4 allele carriers exhibit exacerbated hippocampal damage through impaired lipid metabolism and synaptic repair (Aboouf et al., 2023; Taylor et al., 2024). Additionally, expression differences in mitochondrial and metabolic genes (COQ7, NAPG, ADH6) alter oxidative stress responses and energy homeostasis, further determining hypoxic resilience (Sharma et al., 2024). Epigenetic mechanisms, exemplified by JMJD6-mediated histone demethylation, downregulate synaptophysin and disrupt synaptic development, a pathway implicated in intergenerational cognitive deficits that can be pharmacologically modulated (e.g., by ferulic acid; Li et al., 2023). On the other hand, genetic polymorphisms may explain the evolutionary adaptation. Protective alleles in indigenous high-altitude populations (e.g., Tibetans) mitigate cognitive risks via enhanced oxygen utilization and neuroprotection (Burtscher et al., 2021). These genetic and epigenetic variations collectively explain individual disparities in neurovascular function, inflammatory intensity, and antioxidant defenses under hypoxic stress (Zhang et al., 2022). In addition, epigenetic regulators (JMJD6 inhibitors) may restore synaptophysin expression (Li et al., 2023). Genotype-guided antioxidants could counteract APOE4-associated vulnerabilities. Indigenous genetic adaptations may inform hypoxia-resilience therapeutics (Burtscher et al., 2021).

The physiopathologic mechanisms of high-altitude-induced cognitive impairments are highly complicated and yet fully understood. The above-mentioned mechanisms are not independent. The crosstalk among these mechanisms makes it more intricate. For instance, oxidative stress may contribute to exacerbating neuroinflammation, which in turn impacts synaptic plasticity. These interconnected pathways converge to produce cognitive deficits after exposure to hypobaric hypoxia.

## 3 Animal models of high-altitude-induced cognitive decline

Although scientists have conducted extensive research to uncover the physiopathological processes of high-altitude-induced cognitive decline, effective interventions are still limited. More in-depth fundamental research is needed to elucidate neural and molecular mechanisms, thus promoting advances in our understanding and further treatments in this field. Animal models have been widely used in research on hypoxia-related diseases. The implementation of standardized modeling methodologies facilitates not only the investigation into the underlying mechanisms of these diseases but also advances the development of strategies for preventing and treating hypoxic cognitive decline. To ensure the reliability and reproducibility of research findings, critical measures should be noted throughout model establishment, including appropriate experimental animal selection, standardized surgical procedures, and the application of objective assessment metrics (Xu et al., 2025). It should be noted that some animals originally living in the highlands are ideal animal models to explore adaptive mechanisms. Take high-elevation deer mice (*Peromyscus maniculatus*) for instance, these animals live in a wide geographic region (from Alaska and northern Canada southward to western Panama) and exhibit elevated alveolar ventilation and pulmonary oxygen extraction without the pronounced carotid body hypertrophy characteristic of ventilatory acclimatization to hypoxia (VAH) in low-altitude species. This unique adaptation potentially modulates the hypoxic chemoreflex for sustained high-altitude habitation (Ivy and Scott, 2017).

To focus on physiopathology following high-altitude exposure, this review summarizes the established methodologies and evaluation metrics for animal models employed in published mechanistic studies and drug development research for hypoxia-related cognitive impairment (Table 1). Those animal models were categorized according to the altitude and severity of hypoxia, since these factors may directly affect the potential neuropathological mechanisms progressively. For instance, exposure to an altitude of 1,655 m for 1–5 weeks evokes reliable chronic low-grade inflammation, as evidenced by mild changes in circulating immune cells (Nguyen et al., 2021). Exposure to an altitude of 5,000 m for 1 week induces neuroinflammation and amplified systemic inflammation, along with increased inflammatory cytokines (Liu et al., 2022). Moreover, the percentage of damaged neurons is less than 10% in normoxic conditions, but increases to 15%−30% and more than 50% when exposed to altitudes of 4,280 and 7,620 m (Zhu et al., 2022; Sharma and Tulsawani, 2020). The involved mechanisms of cognitive impairment are discussed in detail under various altitude conditions later. Hopefully, this review will establish a reference framework for fundamental research, drug screening, and therapeutic efficacy assessment in hypoxia-related diseases using animal models.

**Table 1:** Animal models of high-altitude-induced cognitive decline.

**Category**	**Species**	**Altitude (m)**	**Exposure duration**	**Cognitive function**	**Behavioral test for cognition**	**Other behavioral assessment**	**Involved mechanism**	**References**
Mild HAE	Long-Evans rats, Sprague Dawley rats	1,655	1–5 weeks	N/A	N/A	Anxiety- and depressive-like behaviors	Inflammation	PMID: 33891978 (Nguyen et al., 2021)
Mild HAE	C57BL/6 and BALB/c mice	2,300	2 weeks	Memory	Y maze	Motor ability, balance and endurance, visual ability	N/A	PMID: 39755749 (Li et al., 2025)
Mild HAE	Kunming mice	3,000	1 h	N/A	N/A	Locomotion activity	Neuronal damage	PMID: 35724762 (Shi et al., 2022)
Moderate HAE	Long-Evans rats	3,540	10 days	Spatial and visual memory impairment	Object displacement test, object replacement test	N/A	VEGF signaling	PMID: 30687018 (Koester-Hegmann et al., 2018)
Moderate HAE	Sprague Dawley rats	4,200	3 days	Learning and memory	Morris water maze test	N/A	Ultrastructure of hippocampal neurons	PMID: 29106903 (Zhang et al., 2018)
Moderate HAE	Sprague Dawley rats	4,250	8 months	Spatial learning and memory	Morris water maze test	Anxiety-like behaviors	Hippocampal damage	PMID: 34981302 (Zhu et al., 2022)
Moderate HAE	Kunming mice	4,300	15 days	Spatial memory, discriminative ability	Morris water maze test, novel object recognition test	Anxiety, motor coordination	White matter injury	PMID: 36355226 (Liu et al., 2023)
Moderate HAE	C57BL/6J mice	5,000	24 h	Episodic-like declarative memory, spatial memory	Novel object recognition, Morris water maze test	Motor function	Organic cation transporter 1 and P-glycoprotein	PMID: 40308775 (Zhao et al., 2025)
Moderate HAE	Sprague Dawley rats	5,000	7 days	Spatial memory	Morris water maze test	Locomotion activity	Neuroinflammation	PMID: 35427564 (Liu et al., 2022)
Moderate HAE	C57Bl/6J mice	5,000	3 weeks	Fear memory	Fear conditioning paradigm	N/A	Microglia	PMID: 39152179 (Hatch et al., 2024)
Moderate HAE	C57BL/6 mice	5,000	4 weeks	Episodic-like declarative memory, spatial recognition memory	Novel object recognition, Y maze, Barnes maze	General locomotor function	Oxidative stress	PMID: 39362869 (Ma et al., 2024)
Extreme HAE	C57BL/6 mice	6,000	6–24 h	Spatial memory and learning	Morris water maze test	Motor function	Systemic inflammation	PMID: 28433745 (Zhou et al., 2017)
Extreme HAE	C57BL/6 mice	6,000	10 h	Episodic-like declarative memory	Novel object recognition	Locomotor activity	Inflammation	PMID: 28097612 (Li et al., 2017)
Extreme HAE	C57BL/6J mice	6,000	3–7 days	Spatial memory and learning	Morris water maze test	N/A	Oligodendrocyte-neuron interaction	PMID: 40546220 (Lv et al., 2025)
Extreme HAE	C57BL/6 mice	6,000	14 days	Spatial memory and learning	Morris water maze test	N/A	Cold-inducible RNA-binding protein (CIRP) binding to GluRl	PMID: 39315498 (Jiang et al., 2024)
Extreme HAE	C57BL/6J mice	6,000	6 weeks	Episodic-like declarative memory, spatial memory	Novel object recognition, Morris water maze test	Locomotor activity	Gut microbiota	PMID: 39704932 (Zhou et al., 2025)
Extreme HAE	C57BL/6 mice	7,000	24 h	Spatial recognition memory	Y maze	Locomotor activity, motor function	Oxidative stress	PMID: 35723652 (Ma et al., 2022)
Extreme HAE	C57BL/6 mice	7,000	48 h	Spatial memory and learning	Morris water maze test	Anxiety	Microglial phagocytosis	PMID: 37234914 (Wang et al., 2023)
Extreme HAE	C57BL/6J mice	7,000	48 h	Episodic-like declarative memory, spatial memory	Novel object recognition, Morris water maze test	N/A	Mitochondrial division, Microglia-mediated synapse elimination	PMID: 40484993 (Chang et al., 2025)
Extreme HAE	C57BL/6J mice	7,000	72 h	Episodic-like declarative memory, spatial memory	Novel object recognition, Morris water maze test	N/A	Systemic inflammation	PMID: 40430556 (Xia et al., 2025)
Extreme HAE	C57BL/6J mice	7,000	72 h	Episodic-like declarative memory, spatial memory	Novel object recognition, Morris water maze test	N/A	Ferroptosis pathways	PMID: 38944360 (Zhang et al., 2024)
Extreme HAE	Sprague Dawley rats	7,620	1–14 days	Spatial memory and learning	Morris water maze test	N/A	Pl3K/GSK3β/CREB pathway	PMID: 33788269 (Kumar et al., 2021)
Extreme HAE	Sprague Dawley rats	7,620	7 days	Spatial memory and learning	Morris water maze test	N/A	Neurotransmission, neuroplasticity and redox homeostasis	PMID: 32488040 (Sharma and Tulsawani, 2020)
Extreme HAE	BALB/c mice	8,000	3 days	Spatial memory and learning	Eight-arm radical maze	N/A	Oxidative stress, inflammation and apoptosis	PMID: 38103103 (Jing et al., 2024)

HAE, high-altitude exposure; N/A, not applicable.

### 3.1 Mild high-altitude exposure

With the comprehensive consideration of various factors (Bliemsrieder et al., 2022), the altitude is classified into three categories: mild high-altitude exposure (1,500–3,000 m), moderate high-altitude exposure (3,000–5,000 m), and extreme high-altitude exposure (>5,000 m). The altitude thresholds are higher than those adopted in human beings (Su et al., 2024a), since we have noticed that animals, especially rodents (Shi et al., 2022), are more tolerant in the high-altitude environment.

While no universally accepted paradigm delineates altitude-induced functional impairment, robust evidence documents neurocognitive deficits in adults, including impairments in motor control, perception, memory, and behavioral regulation. Even mild altitudes (≥2,500 m) demonstrably delay reaction times and compromise psychomotor performance (Virués-Ortega et al., 2006). But the findings of 1500-3000 m have yielded inconsistent results. Some systematic reviews argue that humans' cognition may not be affected by exposure to 1,500–3,000 m (Su et al., 2024a). Interestingly, several lines of evidence find improved sustained attention and anticipation ability of participants at 15% fraction of inspired oxygen (equivalent to an altitude of 2,700 m, i.e., 114 mmHg atmospheric PO_2_) compared to normoxia (Ramírez-delaCruz et al., 2025), suggesting the potential therapeutic effects of normobaric hypoxia to treat cognitive dysfunction.

There are limited studies using animal models at an altitude of 1,500–3,000 m. Nguyen et al. (2024) demonstrate that after exposure to 1,655 m, a pro-inflammatory state can be induced in Long-Evans and Sprague Dawley rats, evidenced by elevated granulocytes and circulating monocytes. Critically, this hypobaric hypoxia amplifies anhedonia (as assessed in the sucrose preference test) and despair-like behavior (as assessed in the forced swim immobility) in Long-Evans rats, suggesting strain-specific vulnerability to altitude-triggered depression endophenotypes. In contrast, this altitude exposure seems to have no significant effects on anxiety-like defensive behavioral responses (as assessed in the open field test; Rodriguez-Hernandez et al., 2024). Another study established an animal model for hypoxic training using a normobaric hypoxic chamber filled with 16% O_2_ and N_2_, corresponding to an altitude of 2,300 m (Li et al., 2025). This study assessed cognitive changes using the Y-maze test and showed that the maze completion time was much longer in the hypoxia group than the normoxic group, while hypoxia training for 2 weeks contributed to cognitive protection (Li et al., 2025). Shi et al. (2022) reveal that after acute exposure to 3,000 m for 1 h, the exercise tolerance (as assessed in the rotarod test) and locomotor activity (as assessed in the open field test) of Kunming mice are comparable to those of control Kunming mice exposure to normobaric normoxia. Taken together, although some cognitive abilities may be negatively affected by mild high-altitude exposure, more animal studies are needed to confirm the reliable cognitive dysfunction under a high-altitude condition (1,500–3,000 m).

### 3.2 Moderate high-altitude exposure

Under the conditions of moderate high-altitude exposure (3,000–5,000 m), multiple cognitive abilities of humans can be impaired, including perceptual processes, working memory, inhibitory control, etc. (Su et al., 2024a) For example, young adults who migrated to Lhasa, Tibet (3,650 m) for 2–3 years exhibit impaired verbal and spatial working memory relative to low-altitude counterparts (Tao et al., 2024). Hypoxic exposure further disrupts sleep-dependent memory consolidation, evidenced by reduced slow-wave activity and impaired performance (Tesler et al., 2015). Notably, acclimatized lowlanders residing at 4,300 m for ≥12 weeks demonstrate significantly increased prevalence of mild cognitive impairment (Hota et al., 2012). At the altitude cutoff of 5,000 m, the reaction time prolongs in emergency medical service personnel (Falla et al., 2024).

Research utilizing diverse animal models reveals altitude-dependent patterns of cognitive dysfunction under hypobaric hypoxia, with impairment severity and nature influenced by elevation, exposure duration, species, and sex. Exposure to simulating 10,000 ft (3,058 m) versus normobaric normoxia differentially alters the efficacy of antidepressants, including selective serotonin reuptake inhibitors (SSRIs), in rodent models (Kanekar et al., 2018). Elevating to 3,540 m, Long Evans rats exposed during early adulthood (P48–P59, 12 days) show impaired spatial and visual memory alongside increased neuronal apoptosis, despite compensatory increases in neurogenesis and microvasculature (Koester-Hegmann et al., 2018). Enrichment interventions mitigate these deficits via VEGF-dependent pathways. Higher exposures (4,010–4,300 m) induce acute cognitive injury. In C57BL/6J mice, rapid ascent (3-day pretreatment with 1-day exposure at 4,010 m) triggers hippocampal-dependent cognitive dysfunction marked by apoptosis, autophagy dysregulation, and neuroinflammation (Bottenheft et al., 2023). Similarly, Sprague-Dawley rats exposed to 4,200 m for 3 days after pretreatment display learning/memory deficits (prolonged Morris' water maze latency), mitochondrial damage, and reduced PGC-1α/SIRT1 neuroprotection (Zhang et al., 2018). Chronic exposure at 4,300 m for 15 days in adult male mice causes anxiety-like behavior, broad cognitive impairment, and white matter injury due to oligodendrocyte differentiation blockade and reversible myelin damage (Liu et al., 2023). Chronic high-altitude exposure (4,250 m) for 8 months in rats induces hippocampal atrophy, altered mitochondrial energy metabolism, blood-brain barrier disruption, and correlates with spatial memory deficits and anxiety-like behaviors, demonstrably quantified via multimodal MRI (Zhu et al., 2022).

Moreover, several previous studies have established animal models of high-altitude-related cognitive impairment. Specifically, in Sprague-Dawley rats exposed to 5,000 m for 7 days, significant spatial learning and memory impairment occurs in the Morris water maze test, concomitant with blood-brain barrier disruption and TLR4/NF-κB/MMP-9 pathway-mediated neuroinflammation. However, open-field test performance remains unaffected (Liu et al., 2022). Elevating exposure intensity, mice housed at 5,000 m for 1–8 months develop progressive hippocampal-dependent memory deficits alongside anxiety-like behaviors, with multimodal MRI revealing structural, microvascular abnormalities, and white matter alterations (Cramer et al., 2019). These correlate with transcriptomic dysregulation in neuroinflammation/angiogenesis pathways, microglial activation, and demyelination. Notably, 3-week exposure at 5,000 m in male C57BL/6J mice specifically impairs fear memory recall without affecting *ex vivo* hippocampal LTP, while inducing maladaptive microglial responses (enhanced surveillance, altered chemotaxis) that may contribute to enduring neurological deficits (Chang et al., 2025). Pharmacological studies further reveal functional vulnerabilities. Acute hypobaric hypoxia compromises anti-fatigue drug efficacy by downregulating OCT1/P-gp transporters, reducing cerebral drug concentrations, and diminishing improvements in motor coordination and recognition memory (Zhao et al., 2025). Conversely, edaravone administration during 1-month 5,000 m exposure mitigates learning/memory deficits by reducing oxidative stress and neuroinflammation, reversing hippocampal neural stem cell exhaustion via periostin regulation (Ma et al., 2024). Collectively, these models establish that cognitive impairment severity escalates with exposure duration (>1 week) at an altitude of 5,000 m, primarily affecting hippocampal-dependent functions and executive domains, with deficits manifesting behaviorally as anxiety, memory failure, and reduced psychomotor performance.

### 3.3 Extreme high-altitude exposure

At an altitude of 5,000 m on the plateau, atmospheric PO_2_ drops to approximately half of the sea level (about 55–60 mmHg), and the blood oxygen saturation of the human body will significantly drop to about 85%. Short- or long-term exposure to extreme altitudes (>5,000 m) inevitably damages the cognitive abilities of humans and animals. For animal models, a majority of studies choose 6,000 and 7,000 m to investigate the high-altitude-induced cognitive dysfunction.

Research utilizing murine models demonstrates that exposure to 6,000 m altitude induces time-dependent cognitive deficits through distinct but interconnected molecular pathways. In mice exposed for 3–7 days, acute high-altitude cerebral edema (HACE) manifests with significant spatial memory decline and learning deficits, characterized by oligodendrocyte-neuronal transcriptomic dysregulation (Lv et al., 2025). Early (3-day) exposure triggers compensatory PI3K/mTOR upregulation in oligodendrocytes, mitigating ribosomal stress (Rps29-bax axis) and oxidative phosphorylation to support myelin repair. By 7 days, prolonged hypoxia suppresses PI3K/mTOR signaling, inducing apoptosis/autophagy via oxidative phosphorylation dysfunction and exacerbated neuroinflammation from enhanced Tnfrsf21-App interactions between oligodendrocytes and excitatory neurons, correlating with progressive cognitive deterioration (Lv et al., 2025). Extending exposure to 14 days induces pronounced hippocampal-dependent memory impairment, driven by cold-inducible RNA-binding protein (CIRP) depletion (Jiang et al., 2024). CIRP deficiency disrupts AMPA receptor GluR1 trafficking, which diminishes synaptic density and dendritic spine integrity, directly impairing learning consolidation. Parallel studies reveal that systemic inflammation synergizes with hypoxia: WIP1-deficient mice exhibit amplified neuroinflammation, exacerbating motor and cognitive dysfunction, while lipopolysaccharide (LPS) priming before hypoxia rapidly intensifies blood-brain barrier disruption via hippocampal aquaporin-4 accumulation, accelerating edema and cognitive/motor deficits within 24 h (Zhou et al., 2017). Gut-brain axis involvement is further implicated in chronic impairment, where hypoxia-induced intestinal barrier damage permits LPS leakage, activating hippocampal microglia and reducing neurotrophic factors (e.g., glutamate), ultimately compromising spatial memory (Zhou et al., 2025). These models establish 6,000 m as a threshold for rapid and severe neural compromise, with impairments progressing from cellular stress responses to structural synaptic loss, unified by dysregulated protein interactions, inflammatory escalation, and barrier failure.

At 7,000 m, the animals are generally exposed to a high-altitude environment for days, and acute hypobaric hypoxia exposure induces rapid and severe cognitive deficits. In C57BL/6J mice exposed for 24 h, significant spatial learning impairment and motor coordination deficits (rotarod) occur alongside oxidative, blood-brain barrier disruption, and neuronal damage in hippocampal CA1 regions (Ma et al., 2022). Extending exposure to 48 h in wild-type mice induces selective recent memory amnesia without altering anxiety, characterized by synaptic loss in hippocampal CA1 due to microglial M1-polarization and excessive phagocytosis of synapses, mediated by CX3CL1/CX3CR1 signaling activation (Wang et al., 2023). Longer exposures (over 7 days) exacerbate hippocampal-dependent memory dysfunction, involving mitochondrial fission-driven microglial activation and ferroptosis (Ma et al., 2022). Across all durations, core pathological features include dendritic spine reduction, synaptic protein loss, and neuroinflammation. Pharmacological interventions demonstrate mechanistic specificity. Mdivi-1 (mitochondrial division inhibitor) rescues memory by suppressing microglial glycolysis and phagocytosis (Chang et al., 2025). Tetrahydrocurcumin ameliorates oxidative stress and boosts glucose transport (Ma et al., 2022). Cistanche phenylethanoid glycosides (PGS) inhibit ferroptosis via GPX-4/SLC7A11 upregulation (Zhang et al., 2024). And Qi Jing Wan/Diosgenin attenuates synaptic damage and inflammation while modulating PDE4C expression (Xia et al., 2025). Collectively, these models establish 7,000 m as a threshold for profound neural compromise, with cognitive deficits manifesting within 24 h and progressing from oxidative stress and synaptic instability to microglial-mediated synaptic stripping and ferroptosis cell death as central effectors.

Additionally, a few studies have also detected the effects of the highest altitude environment (above 7,000 m) on cognition. At very high altitudes, such as 8,000 m, mice exposed for 3 days exhibit significant spatial memory deficits, quantified using the eight-arm radial maze (Jing et al., 2024). These deficits manifest as increased working memory errors, reference memory errors (RME), total errors (TE), and total time (TT) to complete the task, correlating with observable hippocampal histological damage and apoptosis (Jing et al., 2024). Moving to higher, extreme altitudes (exceeding 8,000 m, though specific height not detailed), rat models exposed to hypoxia show severe derangements in spatial navigation assessed by the Morris water maze (Sharma and Tulsawani, 2020), indicative of substantial spatial memory impairment. This cognitive dysfunction is accompanied by pronounced hippocampal damage, vasogenic cerebral edema, neurotransmitter imbalances, and reduced neuroplasticity markers like BDNF and p-CREB. Across these models over 7,000 m, the core cognitive domain impaired is spatial memory, with the severity intensifying at higher simulated altitudes (Kumar et al., 2021). Interventions like HPN, Ganoderma lucidum aqueous extract, and sodium butyrate were shown to mitigate these specific cognitive impairments and their associated neural pathologies, further validating the models.

It should also be noted that heterogeneous methodologies from various studies may affect the findings regarding hypobaric hypoxia-induced cognitive changes. First, most studies simulated the hypobaric hypoxia environment using an animal decompression chamber (Zhao et al., 2022; Zhang X. et al., 2023), while animals in some studies were fed and housed in the high-altitude area (Chen et al., 2022). Besides, the above findings should also be interpreted with caution, since mice are intrinsically more hypoxia-tolerant than rats. Second, the cognitive performances were determined by different behavioral tests, including the novel object recognition, Morris's water maze test, Y-maze test, etc. Third, the experimental settings of these behavioral tests await being unified and optimized. Most behavioral tests were conducted in the normoxic environment, which would undoubtedly be affected by reoxygenation. To avoid the influence of reoxygenation, behavioral tests are recommended to be conducted in a hypobaric hypoxia environment (Ma et al., 2024), especially for the time-consuming Morris water maze test and Barnes maze test.

## 4 *In vitro* models

**Figure 2:**
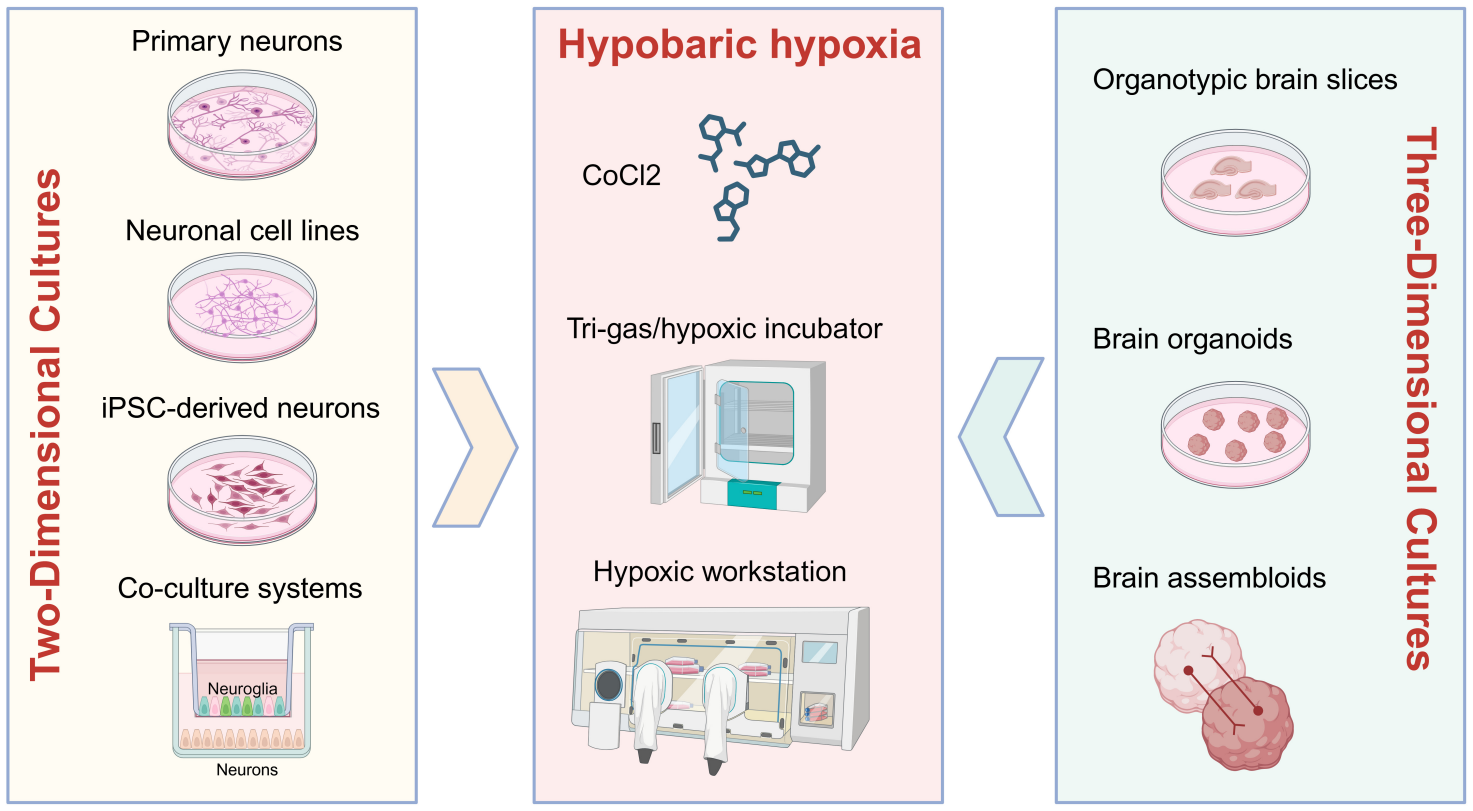
The *in vitro* models for studying cognitive dysfunction induced by high-altitude hypobaric hypoxia. Image was created with BioRender.com with permission.

While human and animal studies have demonstrated the profound effects of high-altitude hypobaric hypoxia on cognitive function, *in vitro* models offer distinct advantages for elucidating the underlying biological mechanisms. Specifically, these models enable researchers to establish highly controlled experimental conditions and precisely select study subjects, thereby facilitating focused and in-depth investigation of specific research questions. As shown in Figure 2, *in vitro* platforms currently employed or potentially applicable for studying plateau environment-associated cognitive impairment include cellular models, co-culture systems, tissue explants, and organoids.

### 4.1 *In vitro* cell models

The neuron, also known as the nerve cell, constitutes the fundamental structural and functional unit of the nervous system. Since neurons play an essential role in the cognitive impairment induced by high-altitude hypobaric hypoxia, they are the most frequently used cells. On the one hand, primary cortical or hippocampal neurons can be isolated from rodent embryos using digestion with 0.25% trypsin. After filtering and purification, primary neurons can be seeded in Neurobasal/B27 medium supplemented with 1% penicillin/streptomycin and 1.25 μM L-glutamine (Zhu et al., 2024). Previous studies have revealed the pharmacological efficacy of diverse drugs in acute high-altitude brain injury by analyzing the survival rates of primary hippocampal neurons (Liu et al., 2024). On the other hand, immortalized neuronal cell lines are also good choices, as they are easier to obtain and cultivate compared to primary cells. Some commonly used neuronal cell lines include human neuroblastoma cells (SH-SY5Y), human cortical neuronal cells (HCN-1A and HCN-2), rat hippocampal neuron cells (H19-7), mouse hippocampal neuron cells (HT22), and mouse motor neuron-like cells (NSC-34).

Moreover, with the fast development of stem cell technology, the induced pluripotent cells (iPSCs) can be easily generated from human somatic cells. Since the human iPSCs capture the genetic background of a given population, they promise to bridge the gap between studies in humans and animal models. Now, iPSCs can be induced to differentiate into neurons, neural glia cells, and other terminally differentiated cell types through exposure to defined combinations of growth factors under specific cell culture conditions (Dolmetsch and Geschwind, 2011). Neurological deficits, including cognitive impairment, are a group of clinical features of chronic mountain sickness. Zhao et al. (2018) have found the altered neuronal excitability, mitochondrial dysfunction, and increased sensitivity to hypoxic stress in iPSC-derived neurons from patients with chronic mountain sickness compared with the control condition. Besides, another study has also established a library of iPSCs derived from the Chinese Han and Tibetan individuals. After differentiation into vascular endothelium, the researchers identify that a hypoxia-dependent enhancer (ENH5) is a key adaptive allele that regulates the EPAS1 gene (encoding HIF-2α) in endothelium (Gray et al., 2025).

Glial cells were traditionally considered the supportive part of the central nervous system. Increasing clues highlight their essential role in neuronal and cognitive function during development and neuropsychiatric disorders. Because of the close connection with the neurovascular unit, astrocytes and microglia are implicated in maladaptive responses to hypobaric hypoxia at high altitude (Chang et al., 2025). Neuropathological examination has found microglia activation and reduced myelination after hypobaric hypoxia exposure (Cramer et al., 2019), suggesting the neuroglia-mediated potential mechanisms. By utilizing a BV2 microglia model exposed to hypoxia, Zhang X. et al. (2025) confirmed the neuroprotective effects of salidroside on blood-brain barrier integrity and cognitive function by inhibiting microglia activation via GSK3β. Yao et al. (2018) generated iPSC-derived astrocytes from highlanders with and without chronic mountain sickness and identified that the Na^+^/H^+^ exchanger (NHE) contributed to the intracellular pH stabilization to render the resistance of astrocytes to hypoxemia challenges.

Currently, *in vitro* cell models typically consist of the cultures of a particular cell type. While these models are widely used to uncover specific molecular pathways, they are not used to investigate the cellular crosstalk among various neuroglia. Therefore, multicellular culture models can be used to mimic *in vivo* biological processes. One of them is the neuron/glia co-culture. Researchers have demonstrated the neuroprotective effects of Antroquinonol by establishing the primary rat neuron/glia coculture systems following exposure to hypoxia and hypoglycemia insults. Considering that neuroinflammation is one of the pathological hallmarks of cognitive impairment induced by high-altitude hypobaric hypoxia, a “tri-culture” model with neuron, astrocyte, and microglia may be more “faithful to explore the interactions in neuroinflammation. Previous evidence has shown the advantages of this tri-culture system over standard mono- and co-cultures (Goshi et al., 2020). Future studies are encouraged to adopt the above *in vitro* cell models in hypoxia incubators or hypoxia-incubated workstations chambers to investigate further the underlying molecular and cellular mechanisms of hypobaric hypoxia exposure.

### 4.2 Organotypic brain slice cultures

*In vitro* cell cultures serve as a valuable tool for elucidating cellular processes within isolated systems and complementing *in vivo* animal studies. Whereas, primary dissociated cultures enable the investigation of homogeneous cell populations, there is an imperative to examine brain cell functionality within three-dimensional (3D) systems (Humpel, 2015). Prior studies have developed numerous matrix materials (type-I collagen, matrigel, porous polystyrene, etc.) to support the 3D culture of human and rodent neural stem/progenitor cells (Murphy et al., 2017). In addition, organotypic brain slice cultures are more able to recapitulate native tissue architecture. These brain slices are typically cut into 100- to 400-μm-thick sections with a vibratome from whole postnatal or adult brains (Humpel, 2015). After recovering for several days, the organotypic brain slices can be cultured *in vitro* for several weeks to facilitate further study (Wu et al., 2020). Specifically, the organotypic hippocampal slice culture represents a highly advantageous model system, which has been previously established to study neurodevelopmental disorders and neurodegenerative diseases. In addition to conventional techniques such as molecular biology and immunohistochemistry, Ca^2+^ imaging can also be carried out to monitor neuronal activity in the brain slice (Wu et al., 2020). Therefore, organotypic brain slice cultures are good *in vitro* models for studying brain diseases induced by exposure to a high-altitude environment.

### 4.3 *In vitro* brain organoid models

Brain organoids are self-assembled (without the need for external scaffolding or meticulous manual guidance) 3D aggregates derived from human pluripotent stem cells (hPSCs), including hiPSCs and human embryonic stem cells (hESCs). These models exhibit cytoarchitectures that resemble those of the human brain and preserve human genetics, representing a novel model to investigate human brain development and neurological disorders (Qian et al., 2019). Compared with conventional 2D cell cultures, there are some advantages to brain organoids. First, brain organoids recapitulate the human brain at the cellular level and extend to encompass tissue architecture and developmental trajectory, thereby providing a unique model for studying human brain development and function, processes often infeasible to investigate directly (Qian et al., 2019). Second, brain organoids inherit the whole set of genetic information of a specific population, making it possible to explore the genetic mechanisms of altitude illness and altitude acclimatization *in vitro*. Third, the organoids can be transplanted into the brain of host animals to study the effects of diverse microenvironments. Previous evidence has shown the functional link between human-animal brain chimeras (Chen et al., 2019). The US Food and Drug Administration (FDA) recently announced plans to reduce animal testing in preclinical drug safety studies by introducing computational modeling and other innovative methods (FDA pushes to replace animal testing, 2025). Undoubtedly, organoids are the most promising innovative methods. The optimist has pointed out that we may see the approval of clinical therapies based on data from organoids in the near future.

Brain organoids have been increasingly used to evaluate drug safety and investigate human brain development and diseases. For instance, our group has identified the developmental neurotoxicity of (2R,6R)-hydroxynorketamine, a metabolite of ketamine, in hESC-derived cerebral organoids (Du et al., 2023). Neurodevelopmental scientists have recognized that exposure to valproic acid during pregnancy increases the risk of autism spectrum disorder in offspring. Leveraging the human brain organoids, we found the decreased neurogenic potential of outer radial glia induced by valproic acid exposure, implying the potential cellular mechanisms of autism spectrum disorder (Zang et al., 2022). Recent work has established the organoid single-cell genomic atlas, which provides a powerful platform for uncovering dynamic gene-regulatory features of human brain development (Kanton et al., 2019). For brain diseases, cerebral organoids have been adopted to reveal the role of apolipoprotein E4 genotype, the strongest genetic risk factor, in Alzheimer's tau pathology (Huang et al., 2022).

Despite the rapid advances, there remain some challenges for expanding the application of brain organoids. First, while brain organoids lack the full complexity of the human brain, the emergence of complex network activity within them has prompted ethical concerns regarding their moral status and the trajectory of research. Engaging ethicists is essential to rigorously evaluate this research using established ethical frameworks (Birtele et al., 2025). Extreme hypoxic conditions should be avoided when simulating the hypobaric hypoxia environment. And it is recommended that ethicists and donors of the organoids participate in the design of study protocols. Second, improving the structural and cellular complexity is urgently needed. By integrating microglia, the neuroimmune organoid model has been successfully established. Other immune cells and, especially, the vascular network are needed to study brain function. Moreover, assembloids based on organoids, such as cortical organoids, hippocampal organoids, hypothalamic organoids, etc., are needed to analyze the crosstalk among various brain regions (Paşca et al., 2022). Third, the culture conditions should be improved. Brain organoids are generally cultured in conventional cell incubators with 5% CO_2_. Relative malnutrition and hypoxia exist inside the organoids, which hinder the studies of long-term environmental exposure using organoids. Currently, we have developed brain organoid models in the setting of high-altitude hypoxia. The pathophysiological data of brain organoids will be released soon.

## 5 Opportunities and challenges

With an increasing number of people visiting highland regions for sightseeing and work, the immigrants' physical and mental health is gaining prominence in the highlands. Tremendous progress has been made by using preclinical models and clinical settings to study pathophysiological changes after exposure to a plateau environment. Population phenotyping studies with cognitive tasks confirm the deleterious effects of hypobaric hypoxia on cognitive function (Aebi et al., 2020). High-density electroencephalogram (EEG) analysis reveals the neural activity patterns and functional connectivity during rapid high-altitude transitions (Xie et al., 2025). Genetic studies identify high-altitude adaptive genetic variants in Tibetans (Guo et al., 2020). For interventions, in addition to pre-acclimatization to hypobaric hypoxia and oxygen inhalation (Shi et al., 2025), dietary and exercise management may help promote adaptation to high-altitude environments (Su et al., 2024b). Moreover, preclinical studies with hypobaric hypoxia chambers examine the neuropathological traits of high-altitude exposure (Zhang et al., 2018; Jiang et al., 2024). Preliminary findings demonstrate abnormal changes in energy metabolism, neuronal autophagy, oxidative stress, and inflammatory response, among others. Animal studies also uncover the potential protective effects of probiotics, traditional Chinese herbs (Xia et al., 2025), and neuroregulators (Ji et al., 2021) on cognitive function in mice chronically exposed to high altitude.

Even though tremendous efforts have been made to understand and protect cognitive functions in the highlands, no relative guideline or expert consensus has been reached. Some limitations should be noted in current studies. First, a key challenge is understanding the hierarchical importance and temporal sequence of the numerous identified pathways. Second, the study designs and study methods can be further improved in participant studies. Specifically, prospective studies with large samples and cutting-edge technologies, such as fMRI and imaging transcriptomics, are needed. Third, a major challenge is bridging the gap between the simplicity of current *in vitro* models and the systemic complexity of high-altitude exposure. Animal studies should be conducted using standard protocols with specific altitudes and oxygen concentrations, which contribute to minimizing the heterogeneity of various studies. Fourth, clinical studies and pre-clinical studies should be considered together when investigating pathological mechanisms and interventions for high-altitude-induced cognitive impairment. The exposure conditions should be consistent or comparable. Fourth, future studies may investigate the potential benefits of mild or short-term exposure to a high-altitude environment. Also, the recovery mechanisms of cognitive impairment are interesting to examine after leaving the high-altitude environment.

## 6 Conclusions

Overall, our understanding of the complexity of cognitive impairment following high-altitude exposure primarily comes from (1) cross-species comparative research exploring psychological/physiological/pathological foundations of human-unique phenotypes, and (2) identification of hypobaric hypoxia-associated genetic variants and interactions. While the potential mechanisms of cognitive changes following exposure to high-altitude hypoxia have been investigated through population studies and preclinical models, significant work remains to be done to elucidate the pathogenesis and identify potential interventions for the future. Population studies involving large participant numbers and the development and generalization of standardized protocols for animal models will facilitate comparisons among investigations. Cutting-edge technologies, including imaging transcriptomics and brain organoids, may allow advanced evaluation of structural and functional impairment in the brain due to altitude. Integrated analyses based on neuroimaging, neuropsychological assessment, animal models, and *in vitro* models will bring new insights into the theoretical relations between hypobaric hypoxia and cognition.
